# Public Health Challenges and Priorities for Kazakhstan

**DOI:** 10.5195/cajgh.2012.30

**Published:** 2012-11-05

**Authors:** Altyn Aringazina, Gabriel Gulis, John P. Allegrante

**Affiliations:** 1 Department of Population Health and Social Sciences, Kazakhstan School of Public Health, Almaty, Republic of Kazakhstan; 2 Unit for Health Promotion Research, University of Southern Denmark, Esbjerg, Denmark; 3 Department of Health and Behavior Studies, Teachers College, and Department of Sociomedical Sciences, Mailman School of Public Health, Columbia University, New York, NY USA

**Keywords:** Central Asia, Kazakhstan, health care, health promotion, population health, public health

## Abstract

The Republic of Kazakhstan is one of the largest and fastest growing post-Soviet economies in Central Asia. Despite recent improvements in health care in response to *Kazakhstan 2030* and other state-mandated policy reforms, Kazakhstan still lags behind other members of the Commonwealth of Independent States of the European Region on key indicators of health and economic development. Although cardiovascular diseases are the leading cause of mortality among adults, HIV/AIDS, tuberculosis, and blood-borne infectious diseases are of increasing public health concern. Recent data suggest that while Kazakhstan has improved on some measures of population health status, many environmental and public health challenges remain. These include the need to improve public health infrastructure, address the social determinants of health, and implement better health impact assessments to inform health policies and public health practice. In addition, more than three decades after the Declaration of Alma-Ata, which was adopted at the International Conference on Primary Health Care convened in Kazakhstan in 1978, facilitating population-wide lifestyle and behavioral change to reduce risk factors for chronic and communicable diseases, as well as injuries, remains a high priority for emerging health care reforms and the new public health. This paper reviews the current public health challenges in Kazakhstan and describes five priorities for building public health capacity that are now being developed and undertaken at the Kazakhstan School of Public Health to strengthen population health in the country and the Central Asian Region.

## Introduction

The Republic of Kazakhstan is one of the former Soviet republics that are now part of the Commonwealth of Independent States (CIS). It has one of the largest and fastest expanding economies in Central Asia, growing an estimated 9% between 2000 and 2007. In 2011, Kazakhstan marked the 20^th^ anniversary of post-Soviet independence by introducing new social policies designed to strengthen its domestic socioeconomic standing and political position in the international community. In addition, the central government of Kazakhstan has prioritized several goals aimed at diversifying the economy beyond its reliance on oil, natural gas, and other extractive industries, decreasing dependence on the government sector, and increasing competitiveness of the state as a whole. A key area of the central government’s interest is the improvement of population health.

Despite strong macroeconomic indicators and considerable progress in building civil society, efforts to democratize its system of higher education and related institutions and modernize infrastructure to support population health, numerous challenges remain in delivering public health services to a population of over 16 million people, 59% of whom now live in the two largest urban centers: Almaty and the capital city of Astana. Although many health status measures show Kazakhstan to be ahead of most nations of the region, Kazakhstan continues to lag behind nations with the size of its economy on several important health and environmental indicators.^1^

This paper briefly reviews the current health status and features of health care in Kazakhstan and describes the current public health challenges. We also present five priorities for public health capacity building that are now being developed at the Kazakhstan School of Public Health to strengthen population health in the country and the Central Asian Region.

## Socio-demographic and Health Status Profile

The population of Kazakhstan is currently estimated (as of 2011) to be 16.5 million, with a growth rate of 1.235%.^2^ The World Bank^3^ has estimated the Gross National Product to be $149.06 billion in 2010, with a per capita gross national income of $6,280, which is below the average of other European Region states.

## Life Expectancy

The World Health Organization (WHO) health profile for Kazakhstan, which is located within the WHO European Region, estimates the average overall life expectancy at birth to be 64 years (59 years for males and 70 years for females), which lags behind the regional average of 75 for both genders.^1^ The trend in life expectancy in Kazakhstan is similar to that observed in the other CIS. Similar to other nations, female life expectancy at birth exceeds that for males by 11 years.

## Causes of Mortality

The WHO estimates that, with only a few exceptions, the rates of mortality due to the main diseases are above the averages for other regional states. According to WHO, noncommunicable chronic diseases (cardiovascular diseases, including diabetes, and cancer) accounted for about 85% of all deaths in Kazakhstan, injuries about 11%, and communicable diseases about 2%.^1^ Cardiovascular diseases accounted for 57% of all mortality.^4^ Infant mortality in 2010 was 29 deaths per 1,000 live births and maternal mortality was estimated in 2008 to be 25 deaths per 100,000 births.

## Infectious and Sexually Transmitted Diseases

Blood-borne diseases such as Hepatitis C and HIV, sexually transmitted infections, and tuberculosis still constitute a challenge for the public health system in Kazakhstan. As of the end of 2010, Kazakhstan reported to the WHO Regional Office for Europe and the European Centre for Disease Prevention and Control a cumulative total of 15,754 HIV and 1,242 AIDS cases, including 881 deaths due HIV/AIDS.^5^ For the year 2010 alone, 1,988 HIV and 256 AIDS cases, with 165 deaths due to HIV/AIDS, were reported. The rate of newly diagnosed HIV infections in 2010 was 12.4 per 100,000 populations. Although Kazakhstan currently has a relatively low prevalence rate of HIV infection, there are a number of factors in place that create the potential for a dramatic increase, including those of migration, injection drug use, commercial sex work, and the marginalization of vulnerable groups.^6^

Kazakhstan has a prevalence rate for tuberculosis (TB) of 198 per 100,000, which, when compared to 63 per 100,000 people for other nations in the WHO European Region, is the highest in the region. While working on a region-wide initiative to improve the effectiveness of the health system in response to TB, Project HOPE initiated an anti-TB strategy by implementing Directly Observed Therapy-Short Course (DOTS) in 1994. Since then, the TB program has expanded to become a region-wide disease management program. In 2009, Project HOPE implemented a five-year, USAID-funded partnership with Population Services International (PSI) to increase access to TB prevention and treatment among at-risk populations in Kyrgyzstan, Tajikistan, Uzbekistan, and Kazakhstan. The initiative provides direct outreach services and training to those populations most at-risk and focuses on increasing TB and HIV knowledge and prevention as well as training for providers on stigma reduction.

## Immunizations

Kazakhstan has been officially considered a poliomyelitis free country by the WHO since 2002, with the national immunization coverage at 98 percent in 2010. Concerning other vaccine-preventable diseases, coverage in 2010 for BCG was at 96%, measles-containing vaccine (MCV) at 98%, and DTP1 and DTP3 were at 99%. Vaccination against 21 infectious diseases is conducted in Kazakhstan. In accordance with the national calendar of prophylactic vaccination, children under two years old are given vaccine treatment against 11 infectious diseases. A National Advisory Committee on Immunization was established in spring 2012 with the aim of being an advisory body to deal with the issues of vaccination and resolve the issues of introduction and application of new vaccines in Kazakhstan. Vaccines certified by the WHO are procured and, moreover, all vaccines undergo laboratory control, tests, hospital control to ensure safety and effectiveness.

## Population Risk Factors

Although the adult risk factors of blood glucose, blood pressure, and the rate of female obesity are higher in Kazakhstan than other countries in the region, male obesity and adult tobacco use is lower. In 2008, the rate of obesity among adult males aged 20+ was 20.2, while adult female obesity was 27.4. According to data collected by the National Center for Healthy Lifestyle Development, the average smoking rate in 1998 was 28%, which had declined to 27% by 2007 and where it has remained as of 2011. Average alcohol consumption has declined from 55% in 1998, to 41.9 % in 2007, to 35.6 in 2011. Adult participation in physical activity was 15.3% in 1998 and 18.6% in 2007; however, in 2011 levels of participation in physical activity declined slightly to 17.6%. Major efforts are now being taken to improve the management and control of hypertension.^7^

## Health Care

Since the post-Soviet era and as the result of reforms, health care in Kazakhstan has developed and evolved into a stable government function that is designed to provide high-quality medical and related pharmaceutical and rehabilitation services to the population. One of the main priorities of the central government of Kazakhstan is the modernization of a high-quality health sector and development of an integrated health care system that utilizes high technology.^8^ The State Health Development Program calls for an intersectoral approach to improving population health and includes benchmarking measures on a wide range of legislative, investment, structural, economic, and personnel indicators. Additionally, one of the main goals of the program is to introduce incentives for people to engage in “self-keeping” behavior and lifestyle changes that can promote and protect health. Thus, the overall goal of the program is to improve health for all citizens of Kazakhstan in an effort to ensure sustainable social and economic development.

The program is also intended to promote dynamic development of the health care system by creating conditions for economic reforms that can improve access to high-quality medical and social services and the provision of prevention programs. This has included development of a new system of social insurance as part of reforms that replaced the Soviet system and are designed to improve access, especially among those in the rural areas where access to care has been problematic,^9^–^11^ and reduce the problem of informal payments to medical personnel that often has been a feature of reimbursement in the provision of medical care.^12^ In addition, the program aims to elevate the professional personnel qualifications of medical specialists and foster development of an equitable health care system that is capable of adapting to market conditions of society. Recent developments along these lines include the creation of a health care legal base, significant increases in health care funding that have allowed for the construction of new, state-of-the-art clinics and hospital facilities, capital repairs and improvement of the technical infrastructure of health facilities, and the introduction of new medical technologies for diagnosis and treatment. As a result of improving the quality and accessibility of health care, some positive trends in the health status of the population related to infectious and other diseases have been achieved. However, the majority of public health parameters remain unsatisfactory.

The State Program’s specific goals for improvement of key indicators by 2015 include:

- increasing life expectancy at birth from 68.4 to 70 years;
- decreasing maternal mortality per 100,000 births from 28.1 to 24.5; and
- decreasing infant mortality per 1,000 births from 16.5 deaths to 12.3.

As in other middle-income countries, the increase in life expectancy is expected to be accompanied by an increase in the number of people living with chronic disease and thus a significant increase in the demand for primary health care is likely to occur.^13^

Even with key elements of the most recent State Program reforms for health care now in place, the health care system continues to be in transition^6^ and significant barriers to implementation have remained.^14^ Kazakhstan’s difficult economic context, lack of resources, the legacy of Soviet-era policies, and the complex and often corrupt political arrangements between national and local authorities are among the challenges that remain to be overcome.^15^ However, there are some positive changes in the health status and health care system of Kazakhstan. For example, health care indicators have continued to improve on most measures as the health care system has modernized. Moreover, the birth rate has increased, together with years of life expectancy, and although the rates of some noncommunicable diseases have risen, the mortality due to cardiovascular diseases has decreased by 1.7%.

## Public Health Challenges

Public health in Kazakhstan is under the direction of the Ministry of Health. Public health efforts are organized and undertaken through a coordinated system of central, regional, and local entities.^16^ There are several key elements of this system. The Committee on Sanitary-Epidemiological Surveillance of the Ministry oversees the “Sanepid” (sanitary-epidemiological) Service, which has organized a network of subdivisions whose role is to conduct disease surveillance, prevent the transmission of communicable diseases, and enact quarantine and other control measures in the event of epidemic outbreaks. The National Program of Health Reform and Development for 2005–2010^17^ outlined an ambitious set of goals for the reduction of infectious diseases through programs that are now being implemented at the national, regional, and local levels. An Interministerial Coordination Committee on AIDS coordinates an inter-agency system of HIV/AIDS centers that are implementing a National Strategic Program on HIV/AIDS Prevention to address the HIV/AIDS epidemic and related diseases. Finally, a National Centre for Healthy Lifestyles focuses on health promotion and disease prevention, including drug abuse and trafficking, alcohol and tobacco use, maternal and child health, nutrition, and environmental health. In addition, primary health care providers, nongovernmental organizations (NGOs) and several international agencies, including USAID, the U.S. Centers for Disease Control and Prevention (CDC), the World Bank, and WHO, provide a range of consultative, programmatic, and other public health services that support domestic public health efforts in Kazakhstan and throughout the region.^16^

Among the most significant public health challenges is the legacy of poor environmental management that has led to two catastrophic environmental health disasters, the effects of which continue to unfold. In the case of the first, a population of approximately 200,000 Kazakhs living in the vicinity of the Semipalatinsk Test Site, located in the steppe region of northeast Kazakhstan, was exposed to large doses of radiation when, starting in the 1940s, the Soviet Union conducted over 400 nuclear weapons tests over the course of four decades. The health and environmental impacts of these tests and the subsequent radiation exposure have become evident in recent decades with the increase in the incidence of cancers and other related diseases.^18^ The second was the environmental disaster that resulted from the draining of the Aral Sea during the 1960s, when Soviet irrigation projects destroyed what was then the fourth largest lake in the world. The destruction of the lake has left the lake bed and surrounding land polluted and the region economically depressed.

Perhaps the most pressing challenges for public health are of organizational, political, and philosophical nature. According to the WHO, the “biggest challenge of the country’s health sector in the domain of public health lies in clarifying, coordinating and streamlining the roles and responsibilities of different agencies responsible for public health and health promotion activities.”^16^ Moreover, related to this is making the promotion of health a core responsibility for all of the government ministries.^19^ The new policy argues for “whole-of-government” and “whole-of-society” approaches that will consolidate the ideas encompassed in *Health in All* policies. This concept emphasizes the need to improve the integration of government activities with health and to reach out beyond government to engage patients and citizens, developing a responsive and inclusive approach to governance for health.^20^ Thus, policy integration across government functions and in intersectoral partnership with the agricultural, education, housing, and transportation sectors will be critical to achieving the goals of public health in Kazakhstan.

Finally, despite recent reforms, young scholars in public health still face significant barriers to mounting successful programs of research that could contribute to improving population health in Kazakhstan and the region. The relative lack of investment in research, the institutional infrastructure necessary to support grants and contracts, and a culture of competitive, peer-reviewed investigational public health science hampers Kazakhstan from achieving breakthroughs in the improvement of population health in a region where the public health needs are critical to advancing the goals of civil society, further economic development, and regional security. The recent attention given to population health and evidence-based practice has catalyzed interest in the region to build the necessary research culture and institutional infrastructure that can support multidisciplinary research to inform policies and practice, not only in Kazakhstan but also across institutions in the region. Such a culture and infrastructure exists in Western Europe, North America, and the United Kingdom, where the benefits of robust national mechanisms for funding research and institutional infrastructures to support competitive procurement of funds is evident in the history of research discoveries that have altered the course of the human condition for the better.

## Priorities for Building Public Health Capacity

Against this backdrop and despite negative trends in many lifestyle indicators and the apparent lack of resources for public health, much has been achieved in the last decade to provide a foundation for the improvement of population health going forward. The establishment of various state programs and many organizations in the field of public health, such as the Kazakhstan School of Public Health (KSPH), has created the essential conditions for the development of a post-Soviet, modern public health movement in the Republic of Kazakhstan.

The central government of Kazakhstan has created a National Coordination Council on Health Protection, the aim of which is the development and maintenance of interactions between central and local executive bodies and international and other organizations for conducting research and programmatic activities on health protection. The Council is a consultative body governed by the Minister of Health, comprising 32 representatives from various ministries, departments, non-governmental scientific institutions, and other organizations that meet quarterly. The main objectives of the Council include the preparation of recommendations and suggestions regarding: 1) performance and maintenance of actions assigned by the programs; 2) improvement of the state policy and normative legislative documents in the area of health protection; and 3) definition of guidelines for health protection of the citizens of Kazakhstan. Thus, the Council is an example of one of the many innovations in public health capacity that is supporting integration of a state health care policy that promotes multi-level, intersectoral decision-making processes concerning the main determinants of health.

The Kazakhstan School of Public Health was established in 1997 in accordance with an agreement with the WHO European Regional office and the Kazakhstan Ministry of Health. The mission of KSPH is to: 1) provide postgraduate education for health care managers, physicians, and other medical specialists; 2) conduct research in public health; and 3) provide expertise and consulting services. One of the objectives of the KSPH academic program is to train public health specialists in a two-year Master’s degree program. Since 2005, 224 students have been prepared with the degree of Master of Science in Public Health; currently, 56 Master of Science students and 15 PhD students are studying at KSPH. KSPH also conducts continuing education for public health specialists with short- and mid-term programs of five-to ten-month durations. More than 12,000 professionals have received professional preparation in public health since 1998.

KSPH is an institutional member of the Asian Pacific Academic Consortium for Public Health (APACPH), the Association of Schools of Public Health in the European Region (ASPHER), and the International Union for Health Promotion and Health Education (IUHPE). Although in recent years new faculties and departments of public health have been established in the country, KSPH remains the flagship professional preparation program in the field of public health in the Central Asian region. As the only well recognized school of public health in the Central Asian Region, KSPH is now pursuing the development of public health capacity in five key priority areas that are critical to advancing public health knowledge and practice in Kazakhstan and the region.

### 1. Implementing the New Public Health

Kazakhstan is implementing a new public health strategy that is guided by a set of core values and principles.^21^–^22^ Health promotion is the cutting edge of this new public health strategy and its theoretical approach and methods are grounded in an ecologic model of health that takes into account cultural, economic, and social determinants and makes a commitment to equity, civil society, and social justice. In 1997, the President of Kazakhstan set out a 30-year welfare strategy for the country in an address titled *Kazakhstan 2030*.^23^ In the address, the President emphasized the importance of public health and health promotion as long-term national priories. The strategy includes the promotion of health and prevention of disease by reducing alcohol, drug, and tobacco use, and improving maternal and child health, nutrition, and the environment. Nevertheless, the current public health model in Kazakhstan is still largely grounded in a biomedical orientation. Key players and decision makers continue to underestimate the importance of public health while they make investments in curative medicine. Thus, there is an urgent need to support the development of public health programs at the local level and encourage an interdisciplinary and intersectoral approach to public health policy and program development. In addition, there is a need to facilitate the increase of knowledge and skills of health promotion specialists in the country and cultivate social responsibility for health.

### 2. Addressing Social Determinants

Recent attention to the social determinants of health^24^ has stimulated a renewed interest in Kazakhstan to improve the social circumstances that are necessary for improved health. Consistent with the WHO’s expectation that Member States in the European Region focus on reducing health inequities that are socially determined, Kazakhstan is currently making progress to improve education, employment, and housing conditions. Efforts to prevent diseases related to poor nutrition, poor sanitation, and poor water supplies also continue to be a priority.^25^ However, more than three decades after the Declaration of Alma-Ata, which was adopted at the International Conference on Primary Health Care convened in Kazakhstan in 1978, improving social circumstances remains a priority that has not fully matured. The newly reconstituted Department of Population Health and Social Sciences at KSPH has been leading the development of work on health economics, health impact assessment, and social determinants of health. Recent Master Classes sponsored with the support of the Ministry of Health have focused on basic principles of public health policymaking, the role of behavioral and social determinants in population health, and the use of economic methods and health impact assessment in evaluating national public health programs. Such activity is designed to convene specialists with different backgrounds from different organizations in order to foster cooperation in finding common ways to solve public health problems that arise from or are related to social determinants.

### 3. Conducting Health Impact Assessment

A third critical priority of KSPH is improving the use of health impact assessment (HIA) to inform policy and benchmark progress.^26^ The powerful hygiene and sanitary system established during the Soviet era provided a good foundation for the introduction of practices like health impact assessment. Yet, the lack of democracy has hindered the full-scale use of the method due to many of same reasons as in other countries of the former Soviet bloc.^27^ HIA is one of the main tools available to public health leaders to implement the *Heath in All* policy approach and is especially relevant for the rapidly developing countries with a high level of economic activity, such as Kazakhstan. Concepts and methods of HIA were first introduced in Kazakhstan in 2005 when KSPH organized and conducted the first summer workshop on the topic for medical and public health professionals. The workshop led to the introduction of a course on HIA into the regular MPH curriculum at KSPH. A second workshop on HIA was conducted in May 2012. An existing Memorandum of Understanding between KSPH and the University of Southern Denmark, Esbjerg, currently provides for ongoing teaching and consultative support in HIA, including the use of HIA in research and as a tool for benchmarking that can inform public health policy and practice. The National Coordination Council on Health Protection seems to be a promising infrastructure within which to situate capacity for HIA, not only at the project level but also the policy level in Kazakhstan.

### 4. Strengthening Scientific Communication and Exchange

A fourth priority is improving public health through scientific communication and exchange. KSPH is focusing on this priority by doing two things. The first involves emphasizing the importance of professional development and, specifically, the value of English as a second language for all of its faculty and students. Fluency in English will be critical to the future development of the public health profession in Kazakhstan. Because the next generation of public health professionals will have to be proficient in conducting searches of the global evidence-base, exchanging knowledge and ideas with colleagues from different countries, and sharing the results of scientific findings and practice in international peer-reviewed journals, English as a second language must become a priority for all professional education in public health. The second involves revitalizing the *Central Asian Journal of Health Service* (*CAJHS*) as a key scientific journal of the region for public health research and practice. *CAJHS* provides many medical professionals from the region a peer-reviewed outlet for publication in the health services. The journal has recently established an online presence and manuscripts are translated into and published in Russian, Kazakh, and English. Expanding the range of published content to include more reports of public health research and commentary, and strengthening the editorial board and peer review capacity of the journal, are key priorities for KSPH, the home of the journal editorial offices.

### 5. Building Public Health Workforce Capacity in Education and Training

The final critical priority is capacity-building in public health professional education and training. KSPH is cooperating with international organizations such as the American International Health Alliance (AIHA), the European Union TACIS Project on Strengthening Environmental Information and Observation Capacity in the Commonwealth Independent States, and other organizations, such as UNICEF, the USAID ZdravPlus Project, and WHO, to achieve institutional reforms and training and education for improving teaching programs on public health and health promotion. In addition, it is working on collaborative research and education projects with the British Council, Council on Health Research for Development (COHRED), Environmental Resources Management (ERM), and the CDC Central Asia Regional Office. KSPH also currently collaborates with several academic institutions, including the Columbia University Global Research Center of Central Asia and the Columbia University schools of education, public health and social work; Ohio University; Semmelweiss University (Hungary); the University at Albany of the State University of New York; and the University of Southern Denmark. KSPH is at the vanguard of efforts to strengthen public health professional education and training and bring its public health curriculum into line with current international standards, including the integration of European and global core competencies and quality assurance standards for health promotion and health education^28^–^29^ and other areas of public health. These efforts include, for example, participating in a program of curriculum reform that is being supported by the Soros Open Society Foundations (Soros Europe Foundation) Higher Education Support Program’s Academic Fellowship Program, and voluntarily undergoing accreditation with the Associations of Schools of Public Health (ASPHER) during 2012–2013. These and other efforts now underway should further the development of educational resources at KSPH and deepen the capacity and professional skills of its academic faculty to conduct scientific research, training courses, and other vital public health functions and services.

## Conclusion

The Republic of Kazakhstan faces many public health challenges. These include the need to improve public health infrastructure, address the social determinants of health, and implement better health impact assessments to better inform health policies and public health practice. In addition, the threats to health created by the unfortunate legacy of Soviet nuclear testing and poor environmental management of natural water resources have continued to endure. In the context of national policy reforms that have placed a high priority on improving the health of the population, the Ministry of Health, the Kazakhstan School of Public Health, and other government and non-governmental entities are responding and making progress with new and fast-developing public health policies and programs to meet the challenges. However, improving public health capacity is critical and will require new incentives and new investments in the system of public health education and training and public health research, if further improvement of population health in Kazakhstan is to be achieved. Continued interest and support of the global public health community— particularly that of WHO—in developing and sustaining international collaborative research and training efforts will be critical and promises to accelerate the process of public health capacity building that is so critical to the future of Kazakhstan and the region.
